# The Effects of Pay for Performance on Disparities in Stroke, Hypertension, and Coronary Heart Disease Management: Interrupted Time Series Study

**DOI:** 10.1371/journal.pone.0027236

**Published:** 2011-12-15

**Authors:** John Tayu Lee, Gopalakrishnan Netuveli, Azeem Majeed, Christopher Millett

**Affiliations:** Department of Primary Care and Public Health, School of Public Health, Faculty of Medicine, Imperial College London, United Kingdom; Yale University School of Medicine, United States of America

## Abstract

**Background:**

The Quality and Outcomes Framework (QOF), a major pay-for-performance programme, was introduced into United Kingdom primary care in April 2004. The impact of this programme on disparities in health care remains unclear. This study examines the following questions: has this pay for performance programme improved the quality of care for coronary heart disease, stroke and hypertension in white, black and south Asian patients? Has this programme reduced disparities in the quality of care between these ethnic groups? Did general practices with different baseline performance respond differently to this programme?

**Methodology/Principal Findings:**

Retrospective cohort study of patients registered with family practices in Wandsworth, London during 2007. Segmented regression analysis of interrupted time series was used to take into account the previous time trend. Primary outcome measures were mean systolic and diastolic blood pressure, and cholesterol levels. Our findings suggest that the implementation of QOF resulted in significant short term improvements in blood pressure control. The magnitude of benefit varied between ethnic groups with a statistically significant short term reduction in systolic BP in white and black but not in south Asian patients with hypertension. Disparities in risk factor control were attenuated only on few measures and largely remained intact at the end of the study period.

**Conclusions/Significance:**

Pay for performance programmes such as the QOF in the UK should set challenging but achievable targets. Specific targets aimed at reducing ethnic disparities in health care may also be needed.

## Introduction

The Quality and Outcomes Framework (QOF), a major pay for performance programme, was introduced into United Kingdom (UK) primary care as part of a new General Practitioner (GP) contract in April 2004. The Framework accounts for approximately one-quarter of a general practice's income [1]. Before the introduction of QOF, most British family doctors were earning a large proportion of their income from capitation payments. This system rewarded family doctors for having a large list of registered patients rather than for the quality of care that they provided [2].

The QOF aims to improve and standardise the quality of primary care and places considerable emphasis on the secondary prevention of cardiovascular disease. As a result, QOF is also considered to have an impact on health care disparities [3]. Clinical quality indicators include the presence of disease registers and standards for the processes of: diagnosis and investigation, referral, clinical monitoring and review, recording and management of risk factors for complications, and treatment and control of risk factors and disease.

Previous literature suggests that physicians may respond to financial incentives but there are also other relevant issues such as their intrinsic motivation [4], [5], [6]. Prior research on the effect of QOF on patients with chronic diseases such as stroke, coronary heart disease and hypertension has not generally taken into account the previous time trends or used patient level data [7]. Campbell et al found that the QOF was associated with accelerated aggregate improvements in diabetes and asthma (but not CHD) management but that these improvements were not sustained over time [8]. However, there remains limited definitive information about the impact of the QOF on practice and patient level disparities in care [9]. We investigated the following research questions: has this pay for performance programme resulted in a step change in the quality of care for coronary heart disease, stroke and hypertension in white, black and south Asian patients? Has this programme reduce disparities in the quality of care for these conditions between these ethnic groups? Did general practices with different baseline performance respond differently to this programme?

## Methods

### Setting and patients

Data used in this study were extracted from a longitudinal primary care record of 220,743 patients registered with 29 family practices in Wandsworth, south London during 2007. The population of Wandsworth is younger than that of England as a whole, with 74% aged<45 years (compared with a national average of 60%). Approximately one in five Wandsworth residents (22%) belong to a minority ethnic group. Of these, 8.8% are black (African or Caribbean) and 4.4% are south Asian (Indian, Pakistani, Bangladeshi). Wandsworth also has a higher than average level of income disparity.

For the purpose of this study, we examined quality of care in all adult patients (≥18 years) diagnosed with stroke, coronary heart disease or hypertension. Patients registered with practices in 2007 with these conditions were identified using an established method which involves searching both diagnostic and management Read and OXMIS codes [10]. Read codes are the clinical classification system used in primary care in the UK; OXMIS codes were used in the past by some general practices but have now been replaced by Read codes. We excluded patients with incorrect data entry and those missing ethnicity coding (13.5% in stroke cohort, 8.1% in CHD cohort, 12.8% in hypertension cohort).

This study was approved by the Wandsworth Local Research Ethics Committee. The committee gave approval for the data to be extracted and analysed without individual patient consent because no patient identifiers were included in the dataset and as such it was anonymised.

See table 1 for the descriptive statistics for patient demographic characteristics.

**Table 1:** Patient characteristics in 2007.

		CHD	Stroke	Hypertension
**Gender**	*Male*	63.07%	51.01%	43.59%
	*Female*	36.93%	48.99%	56.41%
**Ethnicity**	*Black*	9.57%	18.73%	26.26%
	*South Asian*	20.52%	11.86%	13.18%
	*White*	69.19%	68.78%	59.65%
**Age group**	*18*–*45*	1.25%	6.48%	7.66%
	*45*–*54*	6.13%	5.90%	13.08%
	*55*–*64*	18.63%	16.50%	22.77%
	*65*–*74*	33.70%	27.66%	28.30%
	*over 75*	40.29%	43.46%	28.20%
**Co-morbidity**	*no co-morbidity*	17.95%	15.45%	44.08%
	*One co-morbidity*	33.53%	31.90%	30.20%
	*Two co-morbiditiesor more*	48.52%	52.66%	25.72%

### Study variables

The primary outcome measures were mean systolic and diastolic blood pressure and total cholesterol. Each indicator was based on last recorded measurement each year in the electronic record. Our main explanatory variable was ethnicity. Information on ethnic background in primary care is collected from patients during registration, or during the consultation process, using the 2001 UK census classification. Due to small numbers in some of the ethnic groups, we grouped patients into three main categories: white, black, and south Asian. Covariates in the analysis included age, gender, socio-economic status and number of comorbid medical conditions. We assigned socioeconomic status to individual patients based on the postcode of their practice using the Index of Multiple Deprivation 2004. The index of Multiple Deprivation is the most commonly used method of measuring neighbourhood socioeconomic status in the UK and is compiled from a variety of sources, including the 2001 UK census, unemployment, and social security benefit records. We identified additional co-morbid conditions using Read codes in the medical record: asthma, diabetes, depression, heart failure, hypertension, atrial fibrillation, chronic obstructive pulmonary disease, adrenal disease.

### Statistical methods

As previous literature suggests some aspects of patients' health care were already improving before the implementation of pay for performance in UK primary care [11], segmented regression analysis of interrupted time series was used to take into account the previous time trend. This method has recently been widely used in health policy evaluation [8], [12], [13].

Taking into account the multilevel nature of the data (patients being observed many times in the panel, and patients nested at the practice level), a mixed effect multilevel model with two random intercepts was adopted. The model Specification is the following:
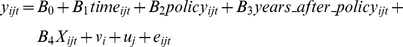
Where 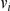,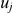 are random intercept for practice level and patient level and are assumed to be independently distributed from the residual error 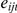. 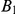 estimates the average change in the outcome measures that occur each year during the study period. 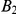 estimates the level change in outcome measures immediately after policy (in year 2005). 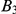 estimates the average change in outcome measures in the years after QOF was introduced (2005–2007). 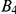 is the vector of estimates corresponding to the vector of covariates that are used to control for patients' heterogeneity. We use the bootstrap method with 2000 replications to estimate the standard error of parameter estimates.

We examined whether the differential effect of QOF for general practices with different baseline performance prior to the implementation of the policy. As some studies have suggested that the differential effect of QOF by practices' with different baseline performance, particularly the worst performers improved at the fastest rate after QOF [14], [15]. For this analysis, we created 3 approximately equal-sized number of GP practices groups based on their baseline performance in year 2003 for each indicator. The same analysis as mentioned before was adopted to examine the differential effect of QOF on clinics with different baseline performance.

This study also investigates whether this pay for performance program reduced disparities among patients in different ethnic groups. We compare differences in blood pressure and cholesterol control between ethnic groups before (year 2003, the year before QOF was introduced) and after (year 2007, the final year of our study) using analysis of covariance (ANOCOVA). For this model, a linear model was adopted which adjusted for age, gender, deprivation, duration of illness, number of co-morbidities and clustering at practice level.

The dataset consists of the historical records (2000–2007) of patients registered with practices in 2007. Some patients might not have complete records throughout each year and we did not capture information on patients with cardiovascular conditions registered with practices during the study period who moved away or died prior to 2007. For example, 71.1% of CHD patients, 61.1% of stroke patients, and 63.8% of hypertension patients have complete record from throughout the study period). To compensate for this, we conducted sensitivity analysis using imputation method and Heckman sample selection model (results in Appendix S1) and compare these results to those from the main analysis.

To increase the power to detect significant predictors of the outcome, the results showed the most parsimonious model which excludes the covariates which are not significant through step-wise back elimination. The standard error was calculated using bootstrapping method with 2000 replications. Statistical analyses were undertaken using STATA version 11.

## Results

**Figure 1:**
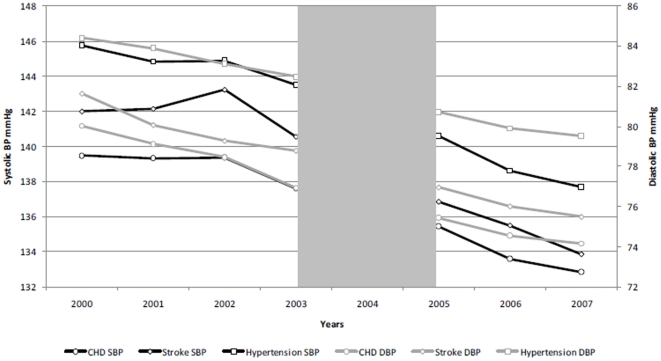
Trends in mean systolic/diastolic blood pressure in patients with CHD, stroke and hypertension.

**Figure 2:**
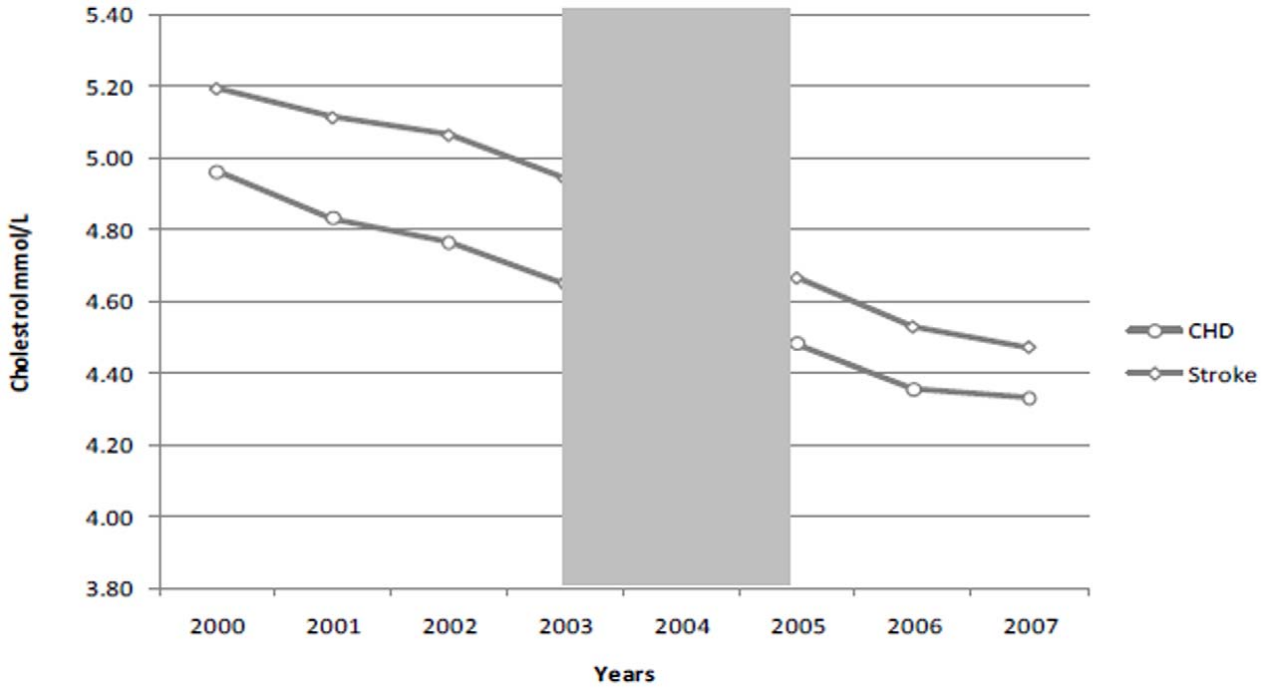
Trends in mean total cholesterol in patients with CHD and stroke.

Our final sample contained 1753 patients with stroke, 2952 patients with coronary heart disease and 15,035 patients with hypertension. In 2007, the mean age of patients with CHD was 68.3 years, 66.9 years for stroke patients and 65.8 years for hypertension. In the CHD cohort, 68.0% were white, 9.8% were black and 21.3% were south Asian. In the stroke cohort, 68.8% of patients were white, 18.5% were black and 11.9% were south Asian. In the hypertension cohort, 59.6% were white, 25.9% were black and 13.6% were south Asian. The average number of co-morbidities for patients with the CHD was 1.2, 1.7 for patients with stroke and 1.0 for the hypertension cohort in 2007. The average duration of illness for CHD is 11.0 years, 9.8 years for stroke, and 9.9 for the hypertension. Trends in mean blood pressure and cholesterol over the study period are presented in Figures 1, 2. The results for the interrupted time series analyses are presented in Table 2.

**Table 2:** Result for systolic/diastolic blood pressure and cholesterol level.

		Systolic				Diastolic				Cholesterol Value			
		Whole population	Black	White	South Asian	Whole population	Black	White	South Asian	Whole population	Black	White	South Asian
**CHD**	**BaselineTrend**	−0.66** (−1.04,−0.28)	−1.12(−2.43,0.19)	−0.82*** (−1.26,−0.38)	0.26(−0.65,1.17)	−1.06*** (−1.28,−0.84)	−1.24***(−1.98,−0.51)	−0.92***(−1.17,−0.66)	−1.57***(−2.08,−1.06)	−0.10*** (−0.13,−0.08)	−0.16***(−0.23,−0.08)	−0.10***(−0.12,−0.07)	−0.11*** (−0.17,−0.06)
	**LevelChange**	−0.81(−2.10,0.49)	−1.23(−5.64,3.17)	−0.67(−2.19,0.84)	−1.39(−4.38,1.61)	−0.32(−1.06,0.42)	−1.95(−4.42,0.52)	−0.40(−1.28,0.48)	0.68(−0.99,2.36)	−0.01(−0.08,0.06)	−0.07(−0.30,0.17)	−0.04(−0.13,0.04)	0.16(−0.01,0.33)
	**TrendChange**	−0.53(−1.09,0.02)	0.63(−1.28,2.53)	−0.36(−1.02,0.29)	−1.77***(−3.05,−0.50)	0.32(−0.00,0.64)	0.83(−0.24,1.90)	0.13(−0.25,0.51)	0.91**(0.20,1.63)	0.02(−0.01,0.05)	0.14***(0.04,0.25)	0.01(−0.03,0.05)	0.01(−0.06,0.08)
**Stroke**	**BaselineTrend**	−0.50(−1.12,0.12)	−0.38(−1.67,0.91)	−0.41(−1.17,0.36)	−1.27(−3.14,0.60)	−0.96*** (−1.31,−0.61)	−0.83**(−1.58,−0.08)	−0.70***(−1.14,−0.27)	−2.92***(−3.92,−1.91)	−0.11*** (−0.15,−0.07)	−0.09(−0.18,0.00)	−0.12***(−0.17,−0.06)	−0.13(−0.28,0.02)
	**LevelChange**	−1.92(−3.89,0.05)	−3.59(−7.89,0.70)	−1.94(−4.35,0.47)	1.14(−4.73,7.02)	−0.38(−1.50,0.74)	−0.77(−3.26,1.73)	−0.98(−2.34,0.39)	4.25***(1.09,7.40)	−0.11(−0.23,0.02)	−0.09(−0.35,0.16)	−0.12(−0.27,0.03)	−0.01(−0.42,0.40)
	**TrendChange**	−0.79(−1.64,0.06)	−0.14(−1.96,1.68)	−1.09** (−2.13,−0.05)	−0.28(−2.80,2.24)	0.26(−0.22,0.74)	0.07(−0.98,1.13)	0.04(−0.55,0.63)	2.03***(0.67,3.38)	0.01(−0.05,0.07)	0.05(−0.07,0.16)	0.00(−0.06,0.07)	0.01(−0.17,0.19)
**Hypertension**	**BaselineTrend**	−0.68*** (−0.85,−0.50)	−0.14(−0.47,0.19)	−0.94*** (−1.17,−0.72)	−0.64**(−1.19,−0.08)	−0.92*** (−1.02,−0.82)	−0.80***(−0.99,−0.60)	−0.87***(−1.00,−0.74)	−1.61***(−1.93,−1.29)				
	**LevelChange**	−1.18*** (−1.76,−0.61)	−1.95*** (−3.07,−0.83)	−1.17*** (−1.91,−0.43)	0.05(−1.66,1.76)	−0.77*** (−1.10,−0.43)	−0.84**(−1.50,−0.18)	−0.95***(−1.37,−0.53)	0.44(−0.54,1.42)				
	**TrendChange**	−0.83*** (−1.08,−0.58)	−1.40*** (−1.88,−0.92)	−0.45** (−0.76,−0.13)	−1.29***(−2.03,−0.54)	0.03(−0.11,0.18)	−0.18(−0.46,0.10)	0.04(−0.14,0.22)	0.66***(0.23,1.09)				

*Notes:*

Wald test was used to test the significance of coefficients

Confidence interval in parentheses,

**P-values at 5% level.

***P-values at 1% level.

### Systolic and diastolic blood pressure

#### CHD Cohort

The baseline trend suggests that systolic blood pressure was decreasing significantly in white patients (0.8 mm Hg per year), but not in black and south Asian patients, with CHD before the introduction of QOF. Diastolic blood pressure was decreasing in all three groups before the introduction of QOF. There was no significant level change in systolic or diastolic blood pressure suggesting that the introduction of QOF did not have an immediate beneficial impact on mean blood pressure control. The trend change for systolic blood pressure suggested a significant reduction during the post-QOF period which exceeded the pre-QOF period among south Asian (1.8 mm Hg per year) but not white or black patients. The trend change for diastolic blood pressure suggested a significant increase during the post-QOF period which exceeded the pre-QOF period among south Asian (0.9 mm Hg per year). There was no significant change in white or black patients.

#### Stroke Cohort

The baseline trend suggests that systolic blood pressure was not decreasing in any group before the introduction of QOF. Conversely, diastolic blood pressure was decreasing significantly in all three groups during this period. There was no significant initial level change in systolic blood pressure in any group. The results for initial level change in diastolic blood pressure suggested that south Asian patients experienced a significant immediate increase (4.2 mm Hg) after the introduction of QOF but there no significant changes among white or black patients. The trend change for systolic blood pressure suggested a significant reduction among white patients (1.1 mm Hg per year) but no change in black or south Asian patients. The trend change for diastolic blood pressure suggested a significant increase among south Asian patients (2.0 mm Hg per year) but no change in black or white patients.

#### Hypertension Cohort

The baseline trend suggests that systolic blood pressure was decreasing significantly in white (0.9 mm Hg per year) and south Asian (0.6 mm Hg per year) patients but not black patients prior to the introduction of QOF. Diastolic blood pressure was decreasing in all three groups during this period. The level change suggested a significant reduction in systolic and diastolic blood pressure in white and black patients, but not south Asian patients, associated with the introduction of QOF. The trend change suggested that there were sustained reductions in systolic blood pressure in all three ethnic groups in the post-QOF period when compared to the pre-QOF period. The trend change for diastolic blood pressure suggested a significant increase among south Asian patients (0.7 mm Hg per year) but no change in black or white patients.

### Total cholesterol

#### CHD Cohort

The baseline trend suggests that total cholesterol was decreasing significantly in all ethnic groups before the introduction of QOF. The results for level changes in each ethnic group did not reach statistical significance. The trend change for total cholesterol suggested a significant decrease among black patients (0.1 mmol/L per year) but no change in south Asian or white patients during the post-QOF period.

#### Stroke Cohort

The baseline trend suggests that total cholesterol was decreasing significantly in white patients but not in black or south Asian patients prior to the introduction of QOF. There were no statistically significant reductions in the level or trend change in any ethnic group.

Trends in mean blood pressure and cholesterol over the study period in are presented in Figures 1a, b.

The results for the effect of QOF on practices with different baseline performance (table 3) suggest that the largest effects of QOF were in the practices with the worst baseline performance. The effect of QOF is very small, even negative, for practices with higher performance in 2003. The coefficient for previous time trends is larger for practices with a better baseline performance, which suggest they had already improved their quality even before the implementation of QOF. As a result of this, the QOF has a very small effect on these practices.

**Table 3:** Effect of QOF by different baseline performance GP clinics.

		Systolic			Diastolic			Cholesterol Value		
		*Upper*	*Middle*	*Lower*	*Upper*	*Middle*	*Lower*	*Upper*	*Middle*	*Lower*
CHD	*Baseline Trend*	−1.03***	0.35	−1.08***	−1.90***	−1.15***	−0.03	−0.10***	−0.13***	−0.06***
	*Level Change*	0.48	−1.39	−2.01	1.49**	−0.05	−2.67***	0.02	0.02	−0.10
	*Trend Change*	−0.03	−1.65***	−0.21	1.31***	0.22	−0.65**	0.04	0.06**	−0.05
Stroke	*Baseline Trend*	−1.56	−0.58	0.93	−1.71***	−0.90***	0.03	−0.20***	−0.09	−0.04
	*Level Change*	−0.94	−0.05	−5.19***	1.28	−0.15	−3.01***	0.19	−0.10	−0.43***
	*Trend Change*	0.88	−0.96	−2.61***	0.92**	0.14	−0.55	0.08	−0.03	−0.01
Hypertension	*Baseline Trend*	−0.89***	−0.87***	−0.21	−1.52***	−0.69***	−0.43***			
	*Level Change*	−0.07	−0.91	−2.49***	0.04	−1.05***	−1.45***			
	*Trend Change*	−0.23	−0.84***	−1.53***	0.82***	−0.25**	−0.67***			

*Notes:*

**at the 5% level and.

***at the 1% level. Wald test was used to test the significance of coefficients.

The first tertile for the clinics with the best baseline performance prior to QOF, the third tertile for the clinics with the worst baseline performance.

Disparities in blood pressure and cholesterol control between ethnic groups before QOF (2003) and at the end of the study period (2007) for the three conditions is shown in table 4. Throughout the study period black patients had highest mean systolic, diastolic blood pressure and cholesterol level compared to other groups. The magnitude of the disparity in risk factor control were attenuated only on few measures (such as systolic and diastolic blood pressure for black patients with hypertension) and largely remained intact at the end of the study period.

**Table 4:** Ethnic Disparities by patients' disease cohorts for each indicator.

	Ethnicity	Systolic			Diastolic			Cholesterol Value		
		2003	2007	Mean Difference(95 %CI)	2003	2007	Mean Difference(95 %CI)	2003	2007	Mean Difference(95 %CI)
CHD	White	137.6	132.8	−4.8 (−6.1, −3.6)	77.2	74.2	−3.0 (−3.7, −2.3)	4.7	4.4	−0.3 (−0.4, −0.3)
	Black	141.5	138.1**	−3.4 (−7.1, 0.2)	79.5**	76.4**	−3.1 (−5.3, 0.8)	4.7	4.5	−0.2 (−0.4, −0.0)
	South Asian	136.4	130.9	−5.5 (−7.9, −3.1)	75.9**	73.5**	−2.5 (−3.8, −1.1)	4.3**	4.1**	−0.2 (−0.4, −0.1)
	All Group	137.8	132.9	−4.8 (−5.9, −3.8)	77.2	74.3	−2.9 (−3.5, −2.3)	4.7	4.3	−0.3 (−0.4, −0.3)
Stroke	White	140.5	133.2	−7.3 (−9.1, −5.6)	78.9	75.5	−3.4 (−4.3, −2.5)	5.1	4.6	−0.5 (−0.6, −0.4)
	Black	141.9	135.2**	−6.8 (−10.2, −3.4)	81.3**	77.4**	−3.9 (−5.9, −2.0)	4.7**	4.4	−0.3 (−0.5, −0.0)
	South Asian	137.8	132.5	−5.3 (−9.9, −0.8)	76.7**	74.9	−1.8 (−4.2, 0.7)	4.7	4.2**	−0.5 (−0.8, −0.2)
	All Group	140.4	133.5	−6.9 (−8.3, −5.4)	78.7	75.1	−3.6 (−4.7, −2.6)	5.0	4.5	−0.5 (−0.6, −0.4)
Hypertension	White	143.7	138.1	−5.7 (−6.2, −5.1)	81.9	79.1	−2.8 (−3.2, −2.4)			
	Black	144.3**	138.2	−6.1 (−7.0, −5.1)	84.3**	81.1	−3.3 (−3.8, −2.7)			
	South Asian	140.8	135.1**	−5.7 (−7.1, −4.3)	81.1**	78.3**	−2.7 (−3.6, −1.9)			
	All Group	143.5	137.7	−5.8 (−6.3, −5.3)	82.5	79.5	−2.9 (−3.2, −2.7)			

*Notes:*

Figures in the table are mean value.

**represents significantly different to white group after adjustment for age, gender, deprivation, duration of illness, number of co-morbidities and practice level clustering at 5% level of significance.

To address the potential attrition bias of the dataset, we have applied two methods (LOCF and Heckman sample selection model) in the sensitivity analysis. The results in sensitivity analysis are similar to those from our main analysis and suggest that they are robust (see appendix table S1 and S2).

## Discussion

By using interrupted time series, we were able to take into account the previous time trends in quality improvement and to identify the impact of QOF on disparities in intermediate health outcomes. Previous research has examined the overall effect of QOF [7], [16], [17], [18], and also for specific disease areas: for example, coronary heart disease [19], [20], [21], hypertension [19], [21], [22], stroke [19], [23] and diabetes [1], [21], [24], [25], [26]. Our findings are broadly consistent with previous research which suggests that QOF was associated with an initial step change improvement in the quality of care but that these improvements appear to flatten out in subsequent years although the pattern of improvement varied between outcome measures. We found that disparities in risk factor control were attenuated only on few measures and largely remained intact at the end of the study period.

The impact of pay for performance on disparities in care is an important concern for designers of these programmes and policy makers [27], [28]. Because QOF did not reward physicians more in deprived areas, QOF might have perpetuated the inverse care law [29], [30], [31]. However, QOF might also have had an effect in terms of diminishing health care inequalities as collective achievement increases those practices with poorer performance catching up in the later years of the implementation of the policy. This phenomenon has been termed the inverse equity hypothesis [18], [32]. Our findings provide some support for the latter explanation as they suggest that the introduction of QOF was associated with an attenuation of disparities in risk factor control between ethnic groups and more rapid improvements in quality in practices performing badly prior to the introduction of this pay for performance programme. However, consistent with previous work our findings suggest that clinically important differences in risk factor control between ethnic groups have persisted three years after the introduction of QOF [18], [20], [21], [22], [33].

Our study has several strengths and limitations. The key strength of this study is that it utilises patient level longitudinal data with near complete coverage of patients in the study area. In addition, this study is also able to adjust for important patient characteristics covariates and look at the effect of QOF on disparities in intermediate health outcomes among different ethnic groups. One of the limitations of the dataset is that it only includes patients registered with practices in 2007 and we lack information on those who died or moved away before this which may cause attrition bias. To address this, we conducted sensitivity analysis under the assumption of both missing at random as well as not at random. These were results are consistent with those from the main analysis which suggests that they are robust.

Another limitation of this study is relatively small sample size, particularly for CHD and stroke, meant that some of our results did not achieve statistical significance due to lack of power. The ITS method relies on the assumption of linearity of the time trend. Based on the descriptive statistics and year by year analysis for the outcome measures, it appears that most of the improvement in risk factor control occurs early in the study period which means that our analysis may have underestimated the effect of QOF.

Our model did not take autocorrelation in the patient-level into account, instead we adopted a mixed effect model (with random effect for both individual level and practice level) to adjust to correlation of the error term within practices. Previous studies have highlighted the importance of taking into account of the correlation in the error term within the same practice [34], [35].

Previous literature suggests that QOF has had an effect on quality improvement for physicians in general; although there may be little or no effect on physicians already achieving the target before the implementation of the policy. Hence, some general practices can achieve the targets without making any additional efforts in improving quality. Rosenthal et al (2005) [36], by looking at a pay-for-performance scheme in California, found that the policy effect is smaller for physicians for those who have already achieved higher performance at baseline compared to those who have had a worse baseline performance. Therefore, they concluded that paying clinicians to reach a common, fixed performance target might produce little gain in quality for the money spent and will largely reward those already operating at a high performance at baseline. This is a particular worrying issue with QOF since many family practices in the UK had already attained high levels of achievement before the QOF was introduced, which indicates that incentives may be too easy to achieve for some practices [7]. Consequently, we may need to implement more challenging targets while also taking into account their cost-effectiveness [37], [38].

Previous studies on the impacts of QOF have generally either used a cross-sectional analysis or used practice level datasets [6]. Therefore, they did not take into account the previous time trends before QOF was introduced or the individual heterogeneity of patients with underlining health characteristics. These studies that have taken into account both of these effects are Campbell et al (2007, 2009) and Millett et al (2008) [8], [35], [39]. However, the results of the first two studies may not be robust because the authors used non-linear projected trajectories to disentangle the previous time trend with the effect of QOF by using only two time points before its implementation. Their analysis was also limited to a relatively small selected sample of patients. The final study examined the impact of QOF on the quality of diabetes management only.

### Conclusions

The results of the study suggest there is a significant positive relationship between the implementation of QOF and risk factor control in people with cardiovascular disease in primary care. However, this effect is mainly attributed to the practices with a worse baseline performance subsequently achieving QOF targets. We found some attenuation of disparities but this was not true across all outcome measures and ethnicities studied. Our findings suggest that pay for performance programmes such as the QOF in the UK should set challenging but achievable targets. Specific targets aimed at reducing ethnic disparities in health may also be needed.

## Supporting Information

Appendix Table S1 **Sensitivity Analysis, results from last observation carried forward method.**
(DOCX)

Appendix Table S2 **Sensitivity Analysis, results from the Heckman sample selection model.**
(DOCX)

Appendix S1 (DOCX)
