# Supplementary material for: The Effects of Pay for Performance on Disparities in Stroke, Hypertension, and Coronary Heart Disease Management: Interrupted Time Series Study
Source: PLoS One. 2011 Dec 15;6(12):e27236. doi: 10.1371/journal.pone.0027236 (PMC3240616; doi:10.1371/journal.pone.0027236)
Supplement: Appendix Table S1 — Sensitivity Analysis, results from last observation carried forward method. (DOCX) [file pone.0027236.s001.docx]

*Appendix Table S1- Sensitivity Analysis, results from last observation carried forward method*

|  | | Systolic | | | | Diastolic | | | | Cholesterol Value | | | |
| --- | --- | --- | --- | --- | --- | --- | --- | --- | --- | --- | --- | --- | --- |
|  |  | Whole population | Black | White | South Asian | Whole population | Black | White | South Asian | Whole population | Black | White | South Asian |
| **CHD** | **Baseline Trend** | -0.60** (-0.96, -0.24) | -0.86 (-2.12, 0.39) | -0.82*** (-1.23, -0.40) | 0.40 (-0.44, 1.23) | -1.01*** (-1.22, -0.81) | -1.15*** (-1.85, -0.46) | -0.91*** (-1.15, -0.66) | -1.38*** (-1.84, -0.91) | -0.10*** (-0.12, -0.08) | -0.15*** (-0.21, -0.08) | -0.09*** (-0.11, -0.07) | -0.10*** (-0.15, -0.05) |
|  | **Level Change** | -0.93 (-2.14, 0.29) | -2.75 (-6.96, 1.47) | -0.54 (-1.97, 0.90) | -1.71 (-4.47, 1.04) | -0.49 (-1.19, 0.20) | -2.68** (-5.02, -0.35) | -0.34 (-1.17, 0.49) | -0.14 (-1.68, 1.40) | -0.01 (-0.08, 0.05) | -0.08 (-0.29, 0.12) | -0.04 (-0.12, 0.04) | 0.12 (-0.02, 0.27) |
|  | **Trend Change** | -0.58** (-1.11, -0.06) | 0.74 (-1.09, 2.58) | -0.40 (-1.02, 0.22) | -1.92*** (-3.12, -0.73) | 0.27 (-0.03, 0.57) | 0.95 (-0.06, 1.97) | 0.09 (-0.27, 0.45) | 0.71** (0.05, 1.38) | 0.01 (-0.02, 0.04) | 0.13*** (0.04, 0.22) | 0.00 (-0.03, 0.04) | 0.00 (-0.06, 0.06) |
| **Stroke** | **Baseline Trend** | -0.44 (-1.01, 0.13) | -0.30 (-1.53, 0.92) | -0.35 (-1.05, 0.35) | -1.20 (-2.90, 0.49) | -0.88*** (-1.20, -0.56) | -0.90** (-1.61, -0.19) | -0.60*** (-0.99, -0.20) | -2.58*** (-3.48, -1.68) | -0.10*** (-0.14, -0.07) | -0.10** (-0.18, -0.02) | -0.11*** (-0.16, -0.07) | -0.06 (-0.19, 0.07) |
|  | **Level Change** | -1.98** (-3.78, -0.17) | -3.43 (-7.48, 0.62) | -2.04 (-4.23, 0.14) | 0.84 (-4.46, 6.13) | -0.65 (-1.67, 0.37) | -0.73 (-3.08, 1.63) | -1.26** (-2.50, -0.02) | 3.10** (0.30, 5.90) | -0.10** (-0.21, -0.00) | -0.03 (-0.25, 0.20) | -0.10 (-0.22, 0.02) | -0.23 (-0.58, 0.12) |
|  | **Trend Change** | -0.83** (-1.61, -0.05) | -0.28 (-2.00, 1.45) | -1.11** (-2.07, -0.15) | -0.34 (-2.66, 1.98) | 0.19 (-0.25, 0.64) | 0.18 (-0.82, 1.19) | -0.07 (-0.61, 0.47) | 1.73*** (0.50, 2.96) | 0.01 (-0.04, 0.05) | 0.05 (-0.05, 0.15) | 0.01 (-0.05, 0.06) | -0.04 (-0.20, 0.12) |
| **Hypertension** | **Baseline Trend** | -0.65*** (-0.82, -0.49) | -0.13 (-0.44, 0.19) | -0.94*** (-1.15, -0.73) | -0.43 (-0.94, -0.08) | -0.87*** (-0.96, -0.77) | -0.75*** (-0.93, -0.56) | -0.83*** (-0.95, -0.71) | -1.42*** (-1.71, -1.13) |  |  |  |  |
|  | **Level Change** | -1.14*** (-1.68, -0.61) | -1.83*** (-2.88, -0.79) | -1.09*** (-1.78, -0.40) | -0.34 (-1.89, 1.21) | -0.88*** (-1.19, -0.57) | -1.00*** (-1.62, -0.39) | -0.97*** (-1.36, -0.57) | -0.12 (-1.01, 0.77) |  |  |  |  |
|  | **Trend Change** | -0.88*** (-1.12, -0.65) | -1.44*** (-1.89, -0.98) | -0.47** (-0.77, -0.17) | -1.54*** (-2.23, -0.85) | -0.06 (-0.19, 0.08) | -0.24 (-0.50, 0.03) | -0.04 (-0.21, 0.13) | 0.40** (0.00, 0.80) |  |  |  |  |

*Notes:*

Wald test was used to test the significance of coefficients. Confidence interval in parentheses, ** P-values at 5% level *** P-values at 1% level
