# Supplementary material for: The Effects of Pay for Performance on Disparities in Stroke, Hypertension, and Coronary Heart Disease Management: Interrupted Time Series Study
Source: PLoS One. 2011 Dec 15;6(12):e27236. doi: 10.1371/journal.pone.0027236 (PMC3240616; doi:10.1371/journal.pone.0027236)
Supplement: Appendix Table S2 — Sensitivity Analysis, results from the Heckman sample selection model. (DOCX) [file pone.0027236.s002.docx]

*Appendix Table S2-Sensitivity Analysis, results from the Heckman sample selection model*

|  | Systolic for CHD cohort | | Diastolic for CHD cohort | | Systolic for Hypertension cohort | |
| --- | --- | --- | --- | --- | --- | --- |
|  | Heckman Coefficient | Selection Coefficient | Heckman Coefficient | Selection Coefficient | Heckman coefficient | Selection coefficient |
| Baseline trend | 0.85*** (1.40, 0.30) | 0.28***(0.26, 0.31) | -1.34***(-1.67, -1.00) | 0.28***(0.26, 0.31) | -0.80***(-1.04, -0.55) | 0.30***(0.29, 0.31) |
| Level change after QOF | -0.83 (-2.39, 0.74) | -0.06 (-0.16, 0.04) | -0.13 (-1.02, 0.76) | -0.06 (-0.16, 0.04) | -1.25***(-1.93, -0.58) | 0.13***(0.08, 0.17) |
| Trend change after QOF | -0.53 (-1.20, 0.14) | 0.14***(0.09, 0.19) | 0.41**(0.02, 0.79) | 0.14***(0.09, 0.19) | -0.66***(-0.95, -0.37) | 0.07***(0.04, 0.09) |
| Female | 1.49*** (0.82, 2.16) | -0.02 (-0.07, 0.02) | -0.09 (-0.47, 0.30) | -0.03 (-0.07, 0.02) | -0.95***(-1.22, -0.68) | 0.07***(0.05, 0.08) |
| Age | 0.20*** (0.17, 0.23) | 0.01***(0.01, 0.01) | -0.18***(-0.20, -0.16) | 0.01***(0.01, 0.01) | 0.19***(0.17, 0.20) | 0.02***(0.01, 0.02) |
| Duration of illness | -0.10***(-0.16, -0.05) | 0.06***(0.06, 0.06) | -0.04**(-0.08, -0.00) | 0.06***(0.06, 0.06) | -0.05***(-0.08, -0.03) | 0.05***(0.05, 0.06) |
| IMD Quintile2 | -3.03***(-4.01, -2.05) | 0.09***(0.02, 0.15) | -0.85***(-1.41, -0.29) | 0.09***(0.02, 0.15) | -2.18***(-2.60, -1.76) | 0.19***(0.17, 0.22) |
| IMD Quintile3 | -1.73***(-2.67, -0.79) | 0.12***(0.06, 0.18) | -0.23 (-0.76, 0.31) | 0.12***(0.06, 0.18) | -1.39***(-1.78, -0.99) | 0.17***(0.14, 0.19) |
| IMD Quintile4 | -1.83***(-2.84, -0.83) | -0.04(-0.10, 0.02) | -0.30 (-0.87, 0.27) | -0.04 (-0.10, 0.02) | -2.32***(-2.76, -1.88) | 0.06***(0.03, 0.08) |
| IMD Quintile5 | -0.96**(-1.93, -0.00) | 0.11***(0.04, 0.17) | -0.22 (-0.77, 0.32) | 0.10***(0.04, 0.16) | -2.16***(-2.59, -1.74) | 0.27***(0.24, 0.30) |
| One co-morbidity | 4.89***(3.91, 5.87) | 0.22***(0.17, 0.28) | 0.80***(0.24, 1.37) | 0.22***(0.17, 0.28) | -1.70***(-2.03, -1.37) | 0.19***(0.17, 0.21) |
| Two co-morbidities or more | 5.98***(5.01, 6.94) | 0.38***(0.32, 0.43) | 0.52 (-0.05, 1.08) | 0.38***(0.32, 0.43) | -2.49***(-2.85, -2.14) | 0.30***(0.28, 0.33) |
| Black | 3.61***(2.55, 4.66) | 0.10***(0.03, 0.17) | 1.91***(1.31, 2.51) | 0.11***(0.04, 0.18) | 1.08***(0.75, 1.40) | 0.11***(0.09, 0.13) |
| Others | 1.59 (-2.92, 6.09) | -0.22 (-0.47, 0.03) | 2.29 (-0.26, 4.84) | -0.22 (-0.47, 0.03) | -4.15***(-5.88, -2.42) | -0.16***(-0.27, -0.06) |
| South Asian | -1.01**(-1.84, -0.17) | -0.08***(-0.13, -0.03) | -1.70***(-2.14, -1.20) | -0.08***(-0.13, -0.03) | -1.95***(-2.38, -1.53) | -0.00 (-0.03, 0.03) |
| Unknown | 0.59 (-7.36, 8.55) | -0.43**(-0.84, -0.03) | 3.26 (-1.24, 7.77) | -0.44**(-0.84, -0.03) | 8.38***(5.62, 11.14) | -0.16 (-0.33, 0.01) |
| rho |  | -1.00(0.06) |  | -0.16 (0.07) |  | -0.06 (0.03) |
| P value for LR test |  | 0.09 |  | 0.04 |  | 0.03 |

*Notes:*

Wald test was used to test the significance of coefficients. Confidence interval in parentheses, ** P-values at 5% level *** P-values at 1% level

The baseline group for IMD quintile is IMD quintile 1; baseline for co-morbidities is patients with no co-morbidity; baseline for ethnic groups is white patients

Bracket in rho is the standard error.
