# Supplementary material for: The Effects of Pay for Performance on Disparities in Stroke, Hypertension, and Coronary Heart Disease Management: Interrupted Time Series Study
Source: PLoS One. 2011 Dec 15;6(12):e27236. doi: 10.1371/journal.pone.0027236 (PMC3240616; doi:10.1371/journal.pone.0027236)
Supplement: Appendix S1 — (DOCX) [file pone.0027236.s003.docx]

**Appendix**

*Appendix 1: QOF indicator*

CHD 5. The percentage of patients with coronary heart disease whose notes have a record of blood pressure in the previous 15 months

CHD 6. The percentage of patients with coronary heart disease in whom the last blood pressure reading (measured in the last 15 months) is 150/90 or less

CHD 7. The percentage of patients with coronary heart disease whose notes have a record of total cholesterol in the previous 15 months

CHD 8.The percentage of patients with coronary heart disease whose last measured total cholesterol (measured in last 15 months) is 5 mmol/l or less

Stroke 5 - The percentage of patients with TIA or stroke who have a record of blood pressure in the notes in the preceding 15 months

Stroke 6: The percentage of patients with a history of TIA or stroke in whom the last blood pressure reading (measured in the previous 15 months) is 150/90 or less

Stroke 7 - The percentage of patients with TIA or stroke who have a record of total cholesterol in the last 15 months

[Stroke 8 - The percentage of patients with TIA or stroke whose last measured total cholesterol (measured in the previous 15 months) is 5mmol/l or less](https://mqi.ic.nhs.uk/IndicatorDefaultView.aspx?ref=1.09.06.04)

Hypertension 4 – The percentage of patients with hypertension in whom there is a record of the blood pressure in the past 9 months

Hypertension 5 – The percentage of patients with hypertension in whom the last blood pressure (measured in last 9 months) is 150/90 or less

*Appendix 2: Sensitivity Analysis*

In our dataset, some of dependent variables were missing from patients’ records. To compensate, we also conducted two sensitivity analyses based on the assumption of both missing at random and not at random.

If the drop-outs are missing at random (MAR), they can be ignored, and the only issue at hand is the loss of efficiency and power with fewer observations. In this case, last observation carried forward (LOCF) could be a suitable method for imputation. This method is also suitable in our situation considering most of the outcome measures are biological measure and results obtained by adopting this method is more likely (but not certain) to be conservative. Besides, LOCF is a preferred method considering it likely (but not certain) to be conservative.

The results can be found in appendix table S1. The coefficients using LOCF are consistent with original ones for both the CHD and the stroke patients. The only exception is with the systolic blood pressure measurement. QOF has a stronger effect for CHD patients and a smaller effect for patients with stroke. However, since the signs of the coefficients from the sensitivity test are still consistent with original findings, we conclude that using LOFC has only minimal effects on our original findings.

If the item non-response is not at random (MNAR), estimations will be inconsistent. To deal with this problem, a sample selection model was adopted which was first introduced by Heckman (1979). The Heckman selection model consists of two parts. The first is the outcome equation, $y_{ijt}=X_{ijt}B+u_{ijt1}$. The second is a selection equation which is a probit-type equation used to predicts whether or not somebody responds: $Z_{ijt}=W_{ijt}r+u_{ijt2}$. The dependent variable is observed if $Z$ >0. Where $y$ are the outcome measures, $X$ are the covariates in the outcome equation, $W$ are the covariates in the selection equation. In these equations, rho (ρ) is the correlation between two error terms i.e. corr$(u_{ijt1,}u_{ijt2})$=ρ. In our analysis, we used full information maximum likelihood (FIML) without exclusion restriction to estimate the model, therefore the same regressors were used in both equations ($X$ is identical to $W$).

We can use t statistic and Likelihood-ratio (LR) test as a test of $H_{0}:\rho=0$. Under the $H_{0}$, there is no sample selection problem. Selection bias would not be a problem if the estimated correlation coefficient, rho (ρ), is not statistically significant and LR test does not reject independence of the two error terms.

For most of the dependent variables, the results suggest that attrition bias is not a problem. The only exception is with the systolic and diastolic measurement for CHD patients and systolic measurement for hypertension patients. Their results can be found in appendix table S2. The coefficients from the Heckman sample selection model are consistent with the original results, except for a slightly smaller effect for systolic measurement after controlling for sample selection bias in the CHD group and a slightly higher level change in the systolic measurement after QOF in the hypertension group. Therefore, our original estimations in the main text are robust with those from sensitivity analysis under the assumption of both MAR and MNAR.
